# Acrylamide Impacts on Black Soldier Fly Larvae: Growth, Toxicity, Microbes, and Bioaccumulation Risks for Food/Feed Safety

**DOI:** 10.3390/insects16060585

**Published:** 2025-06-01

**Authors:** Jianwei Hao, Jiahui Yang, Yiru Zhang, Shurong Zhao, Shuang Liu, Wenfeng Hu

**Affiliations:** 1 Department of Biological Science and Technology, Jinzhong University, Jinzhong 030600, China; haojianwei@jzxy.edu.cn (J.H.); yjh200311032025@163.com (J.Y.); zyr15386720929@163.com (Y.Z.); z18535523452@icloud.com (S.Z.); 2 Institute of Loess Plateau, Shanxi University, Taiyuan 030006, China; 3 College of Food Science, South China Agriculture University, Guangzhou 510642, China; wfhu@scau.edu.cn

**Keywords:** *Hermetia illucens*, acrylamide, toxicological effects, bioaccumulation

## Abstract

This study investigated the effects of acrylamide on the growth, neurobehavioral responses, gut integrity, microbial composition, and toxicokinetics of black soldier fly larvae (BSFL). Larvae were exposed to acrylamide-contaminated diets at 0.05, 0.5, and 5 mg/kg (dry weight) to assess dose-dependent impacts. Results revealed that acrylamide exposure delayed larval growth peaks and reduced maximum weights by 6.17–76.01% (12–18 days). Additionally, crawling speed decreased significantly at ≥0.5 mg/kg, indicating neurotoxicity. Trypan blue staining demonstrated dose-dependent midgut damage (2.22% in control vs. 25.56% at 5 mg/kg), correlating with compromised nutrient absorption. Gut microbiota analysis showed enrichment of pathogenic genera (e.g., *Escherichia-Shigella*) and suppression of beneficial taxa (e.g., *Klebsiella*), alongside reduced metabolic and immune-related pathways via Kyoto Encyclopedia of Genes and Genomes (KEGG) analysis. Toxicokinetic modeling revealed bioaccumulation, with bioaccumulation factors (BAF) inversely related to substrate concentration (18.67 at 0.05 mg/kg vs. 2.90 at 5 mg/kg). Elimination half-lives (DT_50_) varied from 3.25 to 8.22 days, suggesting concentration-dependent detoxification efficiency. These findings highlight acrylamide’s multifaceted toxicity in BSFL, emphasizing risks in waste valorization and insect-based feed production. This study underscores the need for substrate safety protocols to ensure sustainable applications of BSFL in the circular bioeconomy.

## Simple Summary

This study evaluated acrylamide’s multifaceted toxicity in black soldier fly larvae. Dietary exposure induced dose-dependent growth retardation and neurobehavioral impairments, including delayed developmental milestones and reduced motor activity. Acrylamide compromised gut integrity, triggering dysbiosis characterized by the enrichment of pathogenic taxa and depletion of beneficial symbionts, alongside the suppression of metabolic and immune pathways. Toxicokinetic modeling revealed bioaccumulation potential inversely linked to exposure concentrations, with prolonged elimination kinetics indicating impaired detoxification capacity. These findings highlight acrylamide’s risks in disrupting BSFL physiology and ecological functionality, emphasizing the necessity for substrate safety controls in insect-based bioremediation systems.

## 1. Introduction

Frying represents a prevalent cooking approach employed in both fast food and home cooking. While it imparts appealing flavors and textures, the frying process can lead to alterations in the nutrient composition of foods and the release of harmful substances. The Maillard reaction, which occurs during frying, generates acrylamide, a hazardous contaminant frequently detected in fried foods, especially those rich in carbohydrates such as fried potatoes [1]. For people of all ages around the world, from infants to the elderly, exposure to acrylamide from fried foods is a common occurrence [2]. Acrylamide has been identified as a substance that can cause developmental genotoxicity, neurotoxicity, and potentially carcinogenicity. Extensive human and animal studies involving dogs, cats, guinea pigs, rabbits, and rodents have shown that acrylamide exhibits neurotoxicity at doses ranging from 0.5 to 50 mg/kg/day [3,4,5]. Acrylamide is also toxic to insects: a recent study demonstrated that chitosan–alginate-encapsulated *Lactobacillus fermentum* could ameliorate the acrylamide-induced impairment of locomotor activity in *Drosophila* [6]. Currently, numerous studies are being conducted to explore the neurological effects of acrylamide and its underlying mechanisms. However, despite substantial research efforts using various experimental models, several fundamental questions remain unanswered [7]. Recently, emerging evidence has indicated that alterations in the gut microbial profile might play a pivotal role in the development of neurological diseases [8]. Given the known neurotoxicity of acrylamide, some studies have been initiated to investigate the potential link between microbiota–gut–brain axis signaling and acrylamide-induced neurotoxicity. It is hypothesized that acrylamide can disrupt the gut barrier, allowing bacterial metabolites to trigger microbiota–gut–brain axis communication. This disruption leads to a systemic inflammatory response, which, in turn, damages the blood–brain barrier. Ultimately, this process can result in neuronal degeneration and injury [7]. Moreover, research has demonstrated that dietary acrylamide can modify the composition of the gut microbiota. For instance, it has been reported that acrylamide exposure increases the susceptibility of mice to Salmonella Typhimurium infection. Additionally, acrylamide exposure can cause a significant reduction in body weight and severe damage to the intestinal villi [9]. In China, the mean acrylamide concentrations in various foods were determined to range from 0.3 to 351.09 μg/kg [10]. The Food and Agriculture Organization (FAO) and the World Health Organization (WHO) have carried out global investigations on the acrylamide levels in fried, deep-fried, and baked foods such as cakes, bread, French fries, and chips. The results indicated that the maximum potential acrylamide level could reach 5399 μg/kg [11]. It is crucial to emphasize that acrylamide exposure through food not only poses a threat to human health but also has potential environmental and other adverse impacts when acrylamide residues are present in kitchen waste.

Globally, over 2.1 billion tons of municipal solid waste are generated annually. However, only slightly more than 16% of this waste is recycled, while more than 46% is discarded [12]. The treatment of food waste using black soldier fly larvae (BSFL) represents an environmentally friendly and cost-effective approach to resource recycling [13]. Besides the nutritional composition of the substrate [12], hazardous components such as heavy metals [14] and antibiotics [15] can also impact the growth of BSFL. Previous research has shown that culturing yellow mealworms under different benzo(a)pyrene concentration conditions led to changes in their growth rate constants [16]. Fan et al. [17] discovered that exposing BSFL to varying concentrations (1–100 mg kg^−1^) of naphthalene, fluorene, phenanthrene, and pyrene during the treatment of polycyclic aromatic hydrocarbon (PAH)-contaminated waste resulted in prolonged larval development time and a reduced relative growth rate. These findings suggest that the growth of BSFL can be affected by potentially harmful substances present in food or generated during food processing. Acrylamide, a common dietary hazard, is well-known for its neurotoxic effects on animals. Moreover, studies have indicated that acrylamide can also affect the gut microbiota of mice [9]. Despite these findings, no research has yet been conducted to investigate whether acrylamide impacts BSFL, and if so, how these impacts vary with different exposure levels. This knowledge gap warrants further investigation, as understanding the effects of acrylamide on BSFL is crucial for the sustainable operation of BSFL-based food waste treatment systems.

In another aspect, it is widely recognized that incorporating insects as a protein source for food and feed can contribute to establishing a more sustainable food production chain and mitigate the adverse impacts associated with the production and consumption of conventional animal feed [18]. Nevertheless, when exploring novel food and feed sources, food safety is a crucial factor that cannot be overlooked. The rationale behind measuring bioaccumulation through dietary substrate intake is applicable not only to the entire food safety chain but also to the circular bioeconomy. An increasing number of research studies are focusing on assessing the safety of using insects as food, with a particular emphasis on the ability of reared insects to bioaccumulate various toxins from their substrates. Metals [19] and mycotoxins [20] are among the most frequently investigated contaminants. For instance, a recent study explored the bioaccumulation of benzo(a)pyrene by yellow mealworms. The results indicated that the larvae retained benzo(a)pyrene levels exceeding the approved food safety standards [16]. Similarly, Ratel and colleagues recently evaluated the bioaccumulation of polychlorobiphenyls (PCBs) using Tenebrio molitor larvae. Their findings showed that after 20 days of incubation, PCBs were transferred to the larvae through feeding [21]. Notably, there is a significant lack of information regarding the potential accumulation of acrylamide in edible insects such as the black soldier fly (BSF). This knowledge gap underscores the urgent need for targeted research to address the use of these insects in food and feed applications.

The aim of this study was to determine the following aspects: (1) the impact of dietary acrylamide exposure on the growth dynamics of BSFL; (2) whether acrylamide exposure leads to neurobehavioral impairments and midgut damage in BSFL; (3) how dietary acrylamide exposure induces changes in the microbial composition of the BSFL gut microbiota, and to uncover the microbial adaptation mechanisms under chemical stress; (4) whether different concentrations of acrylamide contamination result in bioaccumulation in BSFL, and if acrylamide exposure has implications for the circular bioeconomy and food safety. This study aims to bridge this knowledge gap by systematically investigating the effects of acrylamide on BSFL growth, neurobehavior, gut integrity, microbiota, and toxicokinetics, thereby providing critical insights for safe applications in waste valorization.

## 2. Materials and Methods

### 2.1. Experiment Design

Larval eggs were procured from Wuliang Biotechnical Co., Ltd. in Guangzhou, China. The egg hatching procedure was carried out in accordance with the method described by Hao et al. [22]. Briefly, prior to being transferred to a controlled-environment incubator, BSF eggs were incubated with wheat bran in a plastic box. The hatching conditions were set at 28 °C and 75% humidity. After hatching, the larvae were reared in wheat bran for four days.

Based on the acrylamide contamination concentrations reported in previous literature [10,11], three acrylamide contamination levels were selected for this study. The acrylamide concentrations in the feed dry matter were set at 0.05 mg/kg, 0.5 mg/kg, and 5 mg/kg, respectively. A control group without acrylamide was designated as CK. Considering the ratio of water to the standard diet (Gainesville or GV diet, consisting of 50% wheat bran, 30% alfalfa meal, and 20% corn meal) in the larval feed, which was 2 mL of water per 1 g of diet, acrylamide solutions with concentrations of 0.025 mg/L, 0.25 mg/L, and 2.5 mg/L were prepared using deionized water and acrylamide. Acrylamide (#A800658, Standard for GC, purity ≥ 99.8%) was purchased from Maclin Inc. (Shanghai, China). All acrylamide exposure solutions used in this study were freshly prepared before the experiments. These solutions were then mixed with the feed to achieve the targeted final acrylamide concentrations of 0.05 mg/kg, 0.5 mg/kg, and 5 mg/kg in the feed (dry matter). Three replicates were included in each experimental group. For each replicate, 100 g of the GV diet mixed with 200 mL of water or the corresponding acrylamide solution was used as the feed for subsequent trials. To inhibit fungal growth during the experiments, the GV substrate was autoclaved for 20 min before adding the contaminated media.

The hatched larvae were divided into two parts. In the first part, 200 four-day-old larvae were placed in plastic containers and exposed to the CK group, as well as the 0.05 mg/kg, 0.5 mg/kg, and 5 mg/kg acrylamide-contaminated diets. Subsequently, these larvae were utilized for analyzing larval growth, enterotoxicity, neurotoxicity, and gut microbiota. In the second part, the remaining hatched larvae were transferred to the GV diet and reared until the third instar stage (7 days) for use in bioaccumulation experiments.

### 2.2. Acrylamide’s Influence on Larval Growth

For each group, which had three replicates, a round plastic box with a diameter of 9.8 cm and a height of 10 cm was employed as the experimental container. To ensure consistency and minimize initial differences both within and between groups, the 200 four-day-old larvae in each replicate were weighed to determine the starting average larval weight. During the experiment, every two days, 10 larvae were randomly selected from each replicate. These larvae were weighed, and their lengths (from age 8) were measured. Subsequently, they were returned to their respective containers. To assess whether there were statistically significant differences among the treatments, a one-way analysis of variance (ANOVA) and the least-significant difference (LSD) multiple comparison test were conducted using GraphPad Prism software (GraphPad Software Inc., San Diego, CA, USA).

### 2.3. Effects of Acrylamide on Larval Crawling Capacity

In the crawling experiment, the primary objective was to detect neuronal damage during the early developmental stages of larvae. Fifteen third-instar larvae were randomly selected from each concentration of the acrylamide-containing feed and the GV feed. These larvae were placed in a phosphate-buffered saline (PBS) solution to wash off any adhering medium. Subsequently, they were transferred to the petri dish filled with 0.8% agarose gel. The bottom of the petri dish was lined with a sheet of grid paper. After allowing the larvae to acclimatize for 10 min, the crawling traces and the number of body contractions were recorded within a 2-min period. Each concentration was replicated 3 times, and the crawl speed results were averaged. Subsequently, the larvae were returned to their respective containers.

### 2.4. Effects of Acrylamide on Larval Gut

Third-instar larvae were randomly collected from the CK and different acrylamide-contaminated groups. Subsequently, the larvae were washed with PBS and stained with a 0.02% Trypan blue solution at room temperature for 30 min while being shaken on a shaker at 40 revolutions per minute (r/min). After staining, the treated larvae were rinsed with PBS to remove the excess dye. Finally, the staining degree was observed under a stereomicroscope to assess the extent of intestinal cell damage, following the method described by Pappus et al. [23]. In each experimental condition, a total of 90 larvae from three replicates were treated.

The larval gut extraction process was performed following these guidelines, which were consistent with those of a previous study by Hao et al. [22] with little difference: (1) Place the third-instar larva on ice for three minutes; (2) To eliminate any external contaminants, remove the larva and wipe its surface with 70% alcohol for 30 s. Then, rinse it three times with sterile water; (3) In a sterile environment, use sterile, fine-tipped forceps to cut the abdomen of the insect. After removing the entire intestine, rinse it twice with a sterile 0.9% NaCl solution. Then, transfer the entire intestine into a 1.5 mL microcentrifuge tube. To indirectly correlate acrylamide concentration with intestinal damage in *Hermetia illucens* larvae, the proportion of stained individuals was utilized as a surrogate metric.

### 2.5. Effects of Acrylamide on the Larval Gut Microbial

Four treatments (CK, 0.05 mg/kg, 0.5 mg/kg, and 5 mg/kg) were used for the analysis of gut microbial. For each triplicate, about 10 BSF larvae were selected, whose guts were dissected as each larval sample (3 samples per treatment). The larval gut extraction process was similar to the method used for assessing acrylamide-induced enterotoxicity. The difference was that eighteen-day-old larvae from different treatments were collected for the extraction. DNA was extracted using a FastDNASpin Kit (MP Biomedicals Inc., San Diego, CA, USA). The V3–V4 regions of the 16S rRNA genes in the intestinal DNA samples were sequenced and analyzed on an Illumina MiSeq PE300 platform (Majorbio Co., Ltd., Beijing, China). For the amplification reactions, the primer sets 338F (GTGGACTACHVGGGTWTCTAAT) and 806R (GTACTCCTACGGGAGGCAGCA) were employed. First, high-quality sequences were extracted using QIIME2 (2024.2) software following the previously described methods. Then, the sequences were clustered into operational taxonomic units (OTUs) based on 97% similarity. Subsequently, rarefaction curves, diversity indices, and OTU classifications were generated. To conduct non-metric multidimensional scaling (NMDS), create heatmaps, and determine the abundance of bacterial populations, R version 3.6.1 was used. Pairwise Spearman correlations (r) in the R psych package were applied to explore the relationships between genera. Gephi v0.9.2 was utilized to analyze the significant (*p* < 0.05) and strong (r > 0.80 or r < −0.80) relationships identified in the network analysis. The Origin 2021 program was employed to generate other plots.

### 2.6. Acrylamide Bioacumulation Study

Building upon the methodology developed for studying BSFL exposed to acrylamide, two-phase bioaccumulation experiments were conducted to evaluate how BSFL absorbed and subsequently eliminated acrylamide from their bodies [19]. Each of the exposure and elimination stages of the experimental period lasted five days. During the exposure phase, third-instar BSF larvae reared on the GV feed were exposed to acrylamide-contaminated feed. Larvae were sampled for analysis on each day from 1 to 5 days. On day 5, the larvae were transferred to acrylamide-free feed. In the elimination phase, larvae were collected daily for five days. Three independent studies were carried out, with different acrylamide levels (0.05, 0.5, and 5 mg/kg) in the substrates. Additionally, a control group was established, where BSF larvae were fed non-contaminated GV feed throughout the 10-day experiment and sampled three times (on days 0, 5, and 10) under the same conditions. At each sampling interval for all treatment levels, larvae were placed in plastic containers to empty their guts for 12 h for depuration. Subsequently, the BSF larvae were frozen at −20 °C.

#### 2.6.1. Acrylamide Extraction

Before the extraction process, the larvae were freeze-dried. Subsequently, 1 g of the ground sample was added to a 50 mL polypropylene flask. To remove the fat and enhance acrylamide solubility, 20 mL of water (maintained at 50 °C) and 5 mL of n-hexane were added to the flask. The flask was shaken at 250 rpm for 30 min on an orbital shaker and then centrifuged at 3000 rpm for 20 min. After removing the fat layer, 1 mL of Carrez I and 1 mL of Carrez II were added to purify the water layer and precipitate proteins. The flask was shaken again at 250 rpm for 30 min on the orbital shaker and then centrifuged at 3000 rpm for 20 min. After centrifugation, three layers were observed in the flask: the top layer, the intermediate (transparent) layer containing acrylamide, and the bottom layer. The intermediate layer was further purified using 1 mL each of Carrez I and Carrez II. The mixture was shaken at 250 rpm for 30 min on the orbital shaker and then centrifuged at 3000 rpm for 20 min. After that, the clear layer was carefully removed from the flask and purified using a QuEChERS method (Shanghai ANPEL Scientific Instrument Co., Ltd., Shanghai, China). Finally, the purified liquid was dried via evaporation under a nitrogen atmosphere. The resulting residue was dissolved in 2 mL of water, filtered through a 20 μm filter, and then analyzed chromatographically.

#### 2.6.2. Acrylamide Quantification

An HPLC system consisting of an Agilent 1260 liquid chromatograph (LC), CTO-20AC column oven, and EClassical DAD3100 (Dalian Elite Analytical Instruments Co., Ltd., Dalian, China) was utilized to determine the acrylamide content. To optimize the detection of acrylamide, the wavelength was adjusted within the range of 190–240 nm to maximize its signal intensity. The LC Solution software (version 2.5.0.842) was employed for data collection, and the data were processed subsequently. A 250 × 4.6 mm, 4.5-µm stainless Zorbax C18 analytical column (Hewlett Packard, Houston, TX, USA) was used. The LC conditions were established based on previous research as follows: (1) the mobile phase was composed of water/methanol (90:10, *v*/*v*); (2) the UV signal at 197 nm was selected for detection; (3) the injection volume was set at 10 μL, and the flow rate was maintained at 1 mL/min; (4) the column temperature was set at 35 °C. According to previous studies, the limit of quantification (LOQ) under these conditions was 12.5 μg/kg [24]. Acrylamide quantification was achieved by measuring the peak area at the acrylamide retention time. A calibration curve was constructed using acrylamide solutions with concentrations of 50, 100, 200, 400, 800, and 1000 µg/kg. The acrylamide content in the samples was then determined by comparing the measured peak area with the calibration curve.

#### 2.6.3. Data Analysis

For the toxicokinetic investigation of acrylamide in BSFL, a single-compartment model was employed to describe the exposure and elimination kinetics of acrylamide. This model considered the animal as a single compartment, with distinct rates for exposure and elimination. Growth was factored into the predictions of exposure and elimination kinetics because the mass of BSFL increased significantly during the trial. Based on invertebrate descriptions, a growth dilution factor was incorporated into the toxicokinetics models using a growth rate constant (K_growth_) to better account for the growth of BSFL [19,25,26]. K_growth_ was calculated using the logistic growth model in GraphPad Prism (Version 9.3.1).

For the exposure phase, the following equation was used (Equation (1)):

(1) $$Q_{\left(t\right)}=C_{0}+\frac{K_{1}}{K_{2}+K_{growth}}\times C_{exp}\times (1-e^{-(K_{2}+K_{growth})\times t})$$

Q_(t)_ is the acrylamide concentration of BSFL in mg/kg at day t; C_0_ is the background acrylamide concentration of BSFL at t = 0; K_1_ is the accumulation rate constant in gsubstrateg^−1^ BSFLday^−1^; K_2_ is the elimination rate constant in day^−1^; and C_exp_ is the feed concentration.

For the elimination phase, the following equation was used (Equation (2)):

(2) $$Q_{\left(t\right)}=C_{0}+\frac{K_{1}}{K_{2}+K_{growth}}\times C_{exp}\times (e^{-(K_{2}+K_{growth})\times (t-t_{c})}-e^{-(K_{2}+K_{growth})\times t})$$

t_c_ represents the last day of the exposure phase. Other parameters are specified in Equation (1).

The toxicokinetic parameters were derived using non-linear regression in GraphPad Prism, and new equations were defined based on the software model and Equations (1) and (2). The bioaccumulation factor (BAF) was calculated by dividing the acrylamide concentration in BSFL at the end of the exposure period (5d) (C_BSFL_) by the acrylamide concentrations in the environment (C_substrate_) (Equation (3)):

(3) $$BAF=\frac{C_{BSFL}}{C_{substrate}}$$

The half-life values of acrylamide (DT_50_) in BSFL were computed (Equation (4)) as follows:

(4) $${DT}_{50}=\frac{ln(2)}{K_{2}}$$

The visualization of the above data used GraphPad Prism (Version 9.3.1).

## 3. Results

### 3.1. The Influence of Acrylamide on Larval Growth

**Figure 1:**
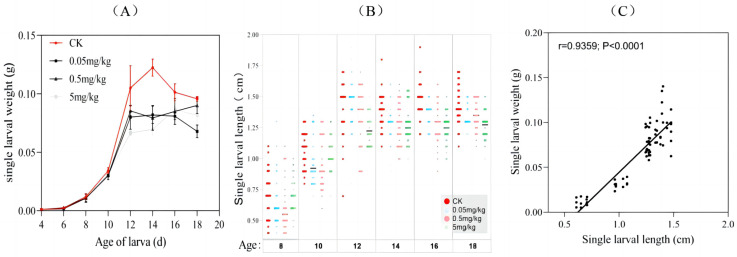
Growth dynamics of BSFL from different treatments: variations in (**A**) larval weight and (**B**) length during bioconversion (age 8–18). (**C**) Pearson’s correlation between larval weight and length.

From day 4 to day 18, the development of the BSFL fed the experimental diets was compared. There was no significant difference in larval weight among the treatments (F3,28 = 0.2376; *p* = 0.8694). Larvae reared on the acrylamide-free diet and the diet with an acrylamide concentration of 0.05 mg/kg reached their maximum weight at 14 days of age, while those on the other diets achieved this at 16 days of age or older. At each day of larval development from 12 to 18 days, individuals on the CK diet weighed 6.17–76.01% more than those on the acrylamide-contaminated diets. Additionally, the larvae fed the acrylamide-free GV diet (mean weight: 0.122 g) had a higher mean weight compared to those fed the acrylamide-contaminated diets (mean weight: 0.082–0.09 g) (Figure 1A).

Since the larvae were too small to measure their length accurately in the early stages, six time points were selected to measure the larval length (Figure 1B). The trend of larval length increase was similar to that of weight. From the fourteenth day of larval development, the CK group had higher mean length values (1.39–1.47 cm) compared to the acrylamide-contaminated group (1.24–1.36 cm) (Figure 1B). Overall, the larvae fed the acrylamide-contaminated diet showed slightly lower larval weight and length compared to the other groups. Moreover, a significant correlation between larval weight and length was observed (correlation coefficient r = 0.9359, *p* < 0.0001). This indicated that acrylamide generally inhibited larval growth, and there was no divergence between these two growth-related indices (Figure 1C).

### 3.2. Effects of Acrylamide on the Crawling Behavior of BSFL

**Figure 2:**
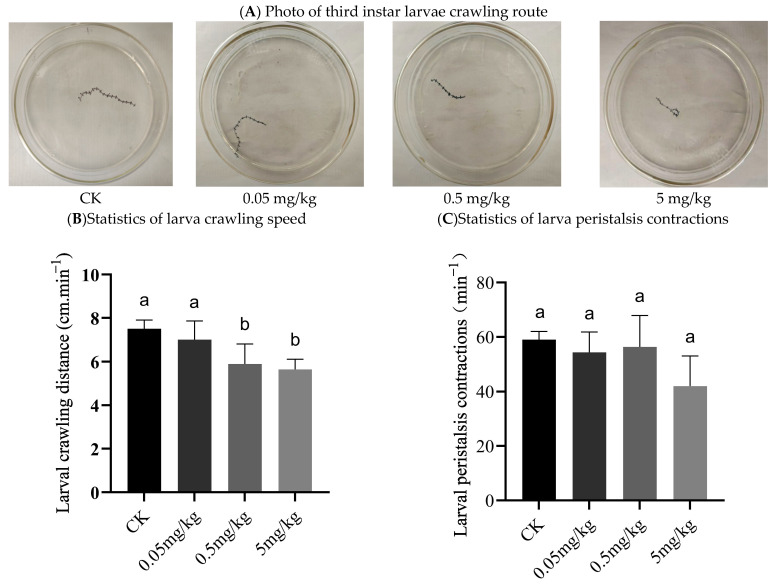
Crawling behavior of third instar larvae: (**A**) photo of third instar larvae crawling route; (**B**) statistics of larva crawling speed; (**C**) statistics of larva peristalsis contractions. Columns marked by the same small letter do not significantly vary (*p* > 0.05).

The results showed that the crawling paths of the larvae were smooth and straightforward in the control group, the 0.05 mg/kg group, and the 0.5 mg/kg group. However, when the acrylamide concentration reached 5 mg/kg, the larvae’s crawling tracks appeared to deviate (Figure 2A). Figure 2B depicts the crawling speed of the larvae. Compared with the control and 0.05 mg/kg groups, the larvae in the 0.5 mg/kg and 5 mg/kg groups crawled at a significantly slower rate. Although there was no significant difference between the acrylamide-contaminated groups and the control group, the mean number of larval peristalsis contractions indicated that the larvae from the acrylamide-contaminated groups (42–56 contractions/min) had fewer contractions than those in the CK group (59 contractions/min) (Figure 2C).

### 3.3. The Impacts of Acrylamide on Larval Gut

**Figure 3:**
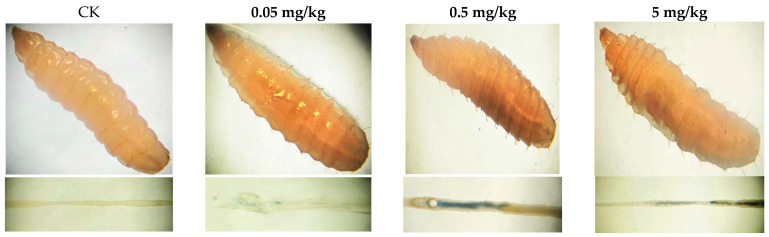
Photo of trypan blue staining of third instar BSFL from different treatments.

In vitro and dissected intestinal images of larvae from different treatments after trypan blue staining are presented in Figure 3. Larvae randomly selected from groups exposed to varying doses of acrylamide showed different levels of staining. Statistical analysis of the staining data revealed that approximately 2.22% of the total number of larvae in the control group had stained midguts, while the percentages of stained larvae in the 0.05 mg/kg, 0.5 mg/kg, and 5 mg/kg groups were 16.67%, 21.1%, and 25.56%, respectively (Table S1). During the staining process, the larvae remained alive until they were photographed and observed in vitro. After the in vitro observation was completed, the gut was dissected and removed for further examination. Both the in vitro and intestinal microscopic results indicated that only the midgut of the larvae was stained. Based on these findings, the midgut cells of BSFL exhibited cell damage at different acrylamide concentrations, and the proportion of damaged cells increased in a dose-dependent manner.

### 3.4. The Impacts of Acrylamide on the Larval Gut Microbial

**Figure 4:**
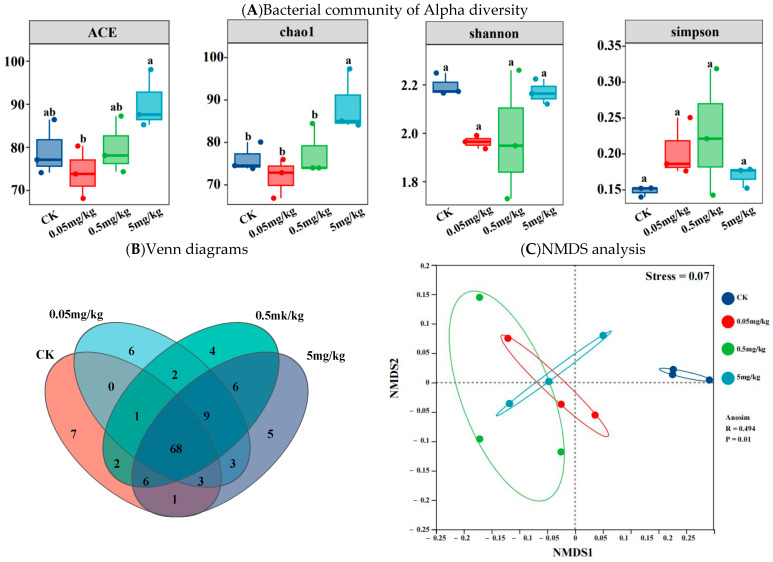
Bacterial community of Alpha diversity (**A**), Venn diagrams (**B**), and NMDS analysis (**C**) based on the Bray–Curtis distance of the BSFL gut for different treatments. Results marked by the same small letter do not significantly vary.

The ACE and Chao1 indices were employed to represent species richness. As depicted in Figure 4A, the ACE and Chao1 indices of the CK group and the acrylamide-contaminated groups at various concentrations differed. However, the differences in ACE values among the CK, 0.05 mg/kg, and 0.5 mg/kg groups were not statistically significant. The samples from the 0.05 mg/kg treatment had the lowest ACE and Chao1 indices, while those from the 5 mg/kg treatment had the highest values. Additionally, in the ACE analysis, the bacterial richness in the 5 mg/kg samples was significantly higher than that in the 0.05 mg/kg samples; moreover, in the Chao1 analysis, it was significantly higher than that in the 0.05 mg/kg, CK, and 0.5 mg/kg samples (*p* < 0.05). The Shannon and Simpson indices were used to represent species diversity in the samples. Although the data varied among the groups, the results indicated no statistically significant differences between them (*p* > 0.05). Venn diagrams showed that 55.3% (68) of the operational taxonomic units (OTUs) detected were common to both the CK and acrylamide-contaminated treatments. These diagrams provided additional evidence of the changes in the gut microbial community structure caused by acrylamide exposure. Compared to the CK group, approximately 20–23 new genera were observed in the guts of BSFL in the different acrylamide-contaminated treatments (Figure 4B). At the genus level, non-metric multidimensional scaling analysis demonstrated that individual samples clustered together. The acrylamide-contaminated samples clustered most closely, while the CK samples showed the greatest separation compared to the acrylamide-contaminated samples (Figure 4C).

**Figure 5:**
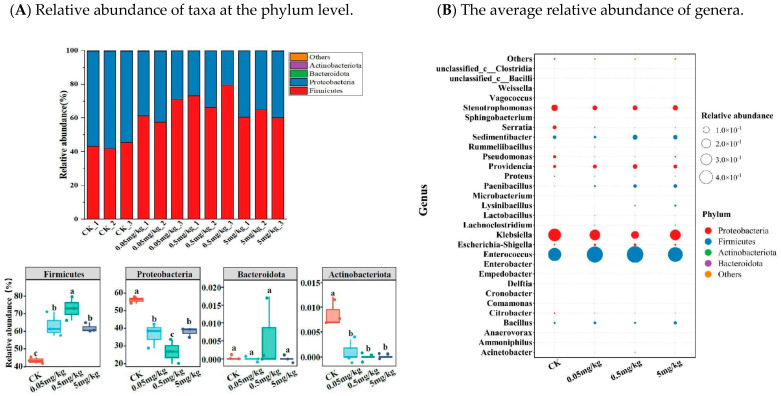
(**A**) Relative abundance of taxa at the phylum level. Bacterial OTUs counted below 0.1% of the total reads were categorized into ‘Others’. (**B**) The average relative abundance (*n* = 3) of genera. Results marked by the same small letter do not significantly vary.

Firmicutes and Proteobacteria, accounting for more than 99% of all microorganisms, were the most prevalent phyla in the larval intestines of all treatment groups. The relative abundance of Firmicutes in the CK was significantly lower (average 44%) compared to the acrylamide-contaminated groups (average 63%, 73%, and 62% for the 0.05 mg/kg, 0.5 mg/kg, and 5 mg/kg groups, respectively; *p* < 0.05). Conversely, the relative abundance of Proteobacteria in CK (average 56%) was significantly higher than that in the acrylamide-contaminated groups (average 36%, 26%, and 38% for the 0.05 mg/kg, 0.5 mg/kg, and 5 mg/kg groups, respectively; *p* < 0.05) (Figure 5A). Moreover, compared to the acrylamide-contaminated groups, the abundance of Actinobacteria in the larval intestine of CK was significantly higher (*p* < 0.05). At the genus level (Figure 5B), acrylamide treatment induced remarkable changes in the gut microbiota composition of BSFL. The enrichment of acrylamide-tolerant genera such as Enterococcus, Escherichia-Shigella, Providencia, Acinetobacter, Lachnoclostridium, Lactobacillus, Paenibacillus, and unclassified_c__Clostridia, along with the suppression of sensitive taxa including Klebsiella, Citrobacter, Anaerovorax, Cronobacter, Microbacterium, Pseudomonas, Serratia, and Stenotrophomonas, indicated potential microbial adaptation mechanisms under chemical stress.

**Figure 6:**
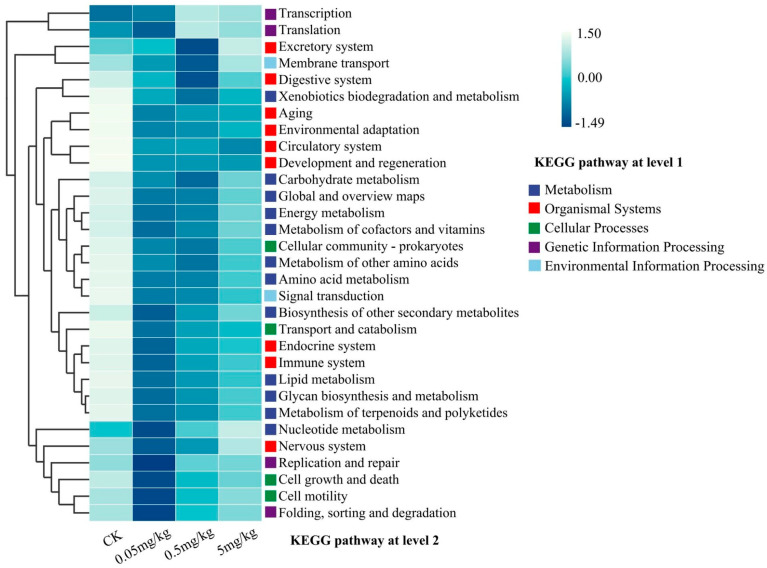
Predictive functional profiling (at the KEGG pathway level 2) of the bacterial communities in the insect gut, analyzed using PICRUSt2 (version 2.2.0) based on the KEGG database. Relative percentage values for the KEGG pathways are represented by color intensity (blue).

The potential functional profiles of the gut bacterial community were analyzed via KEGG pathways. When compared to the CK, acrylamide was found to affect the predicted functional profiling. Specifically, there was a reduction in the predicted organismal systems, encompassing the digestive system, aging-related functions, environmental adaptation, circulatory system, development and regeneration, and immune system (Figure 6). Moreover, acrylamide led to a decline in metabolic functions, such as xenobiotic biodegradation and metabolism, along with nutrient metabolism, including lipid and carbohydrate metabolism. Notably, the influence of acrylamide on KEGG pathways varied according to its concentration. For the majority of the secondary KEGG pathways within the metabolism system, environmental information processing, cellular process, and genetic information processing, the effects exhibited a decreasing trend as the acrylamide contamination concentrations increased (Figure 6 and Figure S1). Furthermore, a correlation analysis based on Spearman’s correlation coefficients was carried out to explore the co-occurrence of the identified predicted functions within the organismal systems cluster and gut bacteria at the genus level. The results indicated a significant negative correlation between Enterococcus and most of these functions. In contrast, Klebsiella, Citrobacter, Cronobacter, Microbacterium, Pseudomonas, Serratia, and Stenotrophomonas were significantly positively correlated with most of these functions (Figure S1A). It is worth noting that Paenibacillus was negatively correlated with axon regeneration.

### 3.5. Acrylamide Bioaccumulation in BSFL

**Figure 7:**
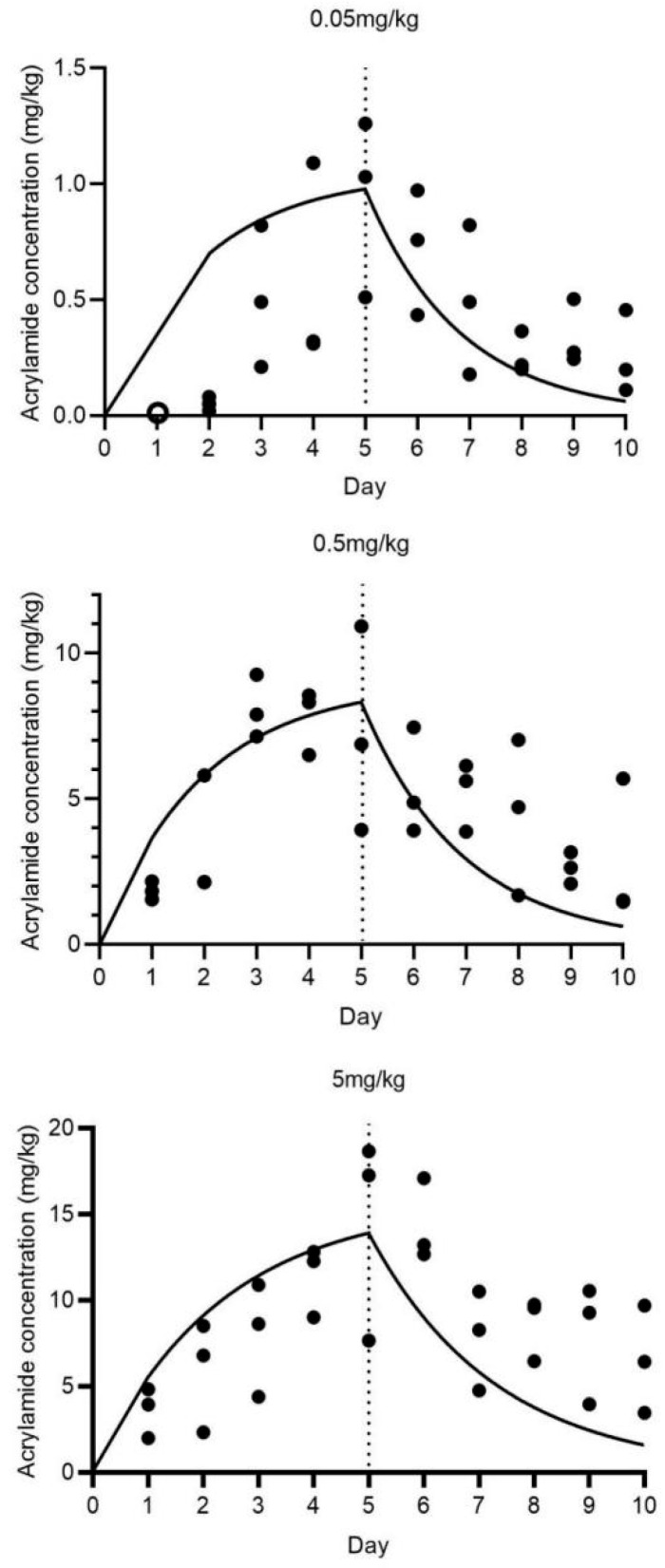
Uptake and elimination kinetics of acrylamide in BSFL exposed to 0.05, 0.5, and 5 mg/kg for 5 days, plus 5 days in a clean substrate. Note: Circle points represent data not considered in the toxicokinetic modeling. Solid lines show the fit of a one-compartment model with growth dilution. The dashed vertical line defines the last day of exposure to the contaminated substrate, ending the uptake and starting the elimination phase of the experiment.

Figure 7 depicts the uptake and elimination kinetics of acrylamide in BSFL exposed to substrate concentrations of 0.05, 0.5, and 5 mg/kg (dry weight). During the uptake phase, BSF larvae demonstrated a time-dependent increase in acrylamide accumulation. For the 0.05 mg/kg treatment, the acrylamide concentration in BSF larvae increased gradually, reaching 0.933 ± 0.384 mg/kg at the end of the uptake phase. Additionally, the body concentrations of BSFL exposed to 0.05 mg/kg were below the detection limit on day 1. A similar trend was observed in the 0.5 mg/kg treatment, with an initially relatively steeper increase. In the 5 mg/kg treatment, although uptake was also apparent (acrylamide concentration at day 5: 14.522 ± 5.988 mg/kg), the rate seemed to deviate from that of the lower-concentration treatments, potentially due to the saturation of uptake mechanisms or other physiological responses. As presented in Table 1, the growth rate constant (K_growth_) for BSF larvae exposed to the 0.05, 0.5, and 5 mg/kg acrylamide-contaminated substrates was 0.3891, 0.3376, and 0.3501 g^−1^, respectively. The uptake rate constant (K_1_) was 11.531, 9.341, and 1.363 g_substrate_$g_{\mathrm{BSFL}}^{-1}$day^−1^, and the elimination rate constant (K_2_) was 0.163, 0.182, and 0.084 day^−1^.

The kinetic bioaccumulation factor (BAF_kinetic_) values were 18.67, 15.46, and 2.90 g_substrate_$g_{\mathrm{BSFL}}^{-1}$, and the half-life for acrylamide elimination (DT_50_) was 3.25, 1.81, and 8.22 days, respectively. These parameters indicate that BSF larvae possess different accumulation and elimination capabilities for acrylamide depending on the substrate concentration. The high BAF_kinetic_ values at lower acrylamide concentrations suggest a relatively strong bioaccumulation potential, while the variation in DT_50_ values reflects the concentration-dependent elimination efficiency.

## 4. Discussion

In 2002, Swedish scientists first detected the presence of acrylamide in heat-processed foods rich in asparagine, which triggered widespread concern [27]. In the same year, two articles confirmed that the Maillard reaction was the primary cause of acrylamide formation in heat-processed foods, raising concerns about acrylamide exposure through normal dietary intake [1,28]. In 2015, the European Food Safety Authority (EFSA) released a survey report on the acrylamide content in 43,419 food products. The results indicated that the average acrylamide content in fried potato products was as high as 1.0 mg/kg, while the highest acrylamide content in coffee reached 4.5 mg/kg. In China, the mean acrylamide concentrations in different foods were found to range from 0.3 to 351.09 μg/kg [10]. Consequently, acrylamide contamination in food ingredients has emerged as a significant issue. Existing research has predominantly concentrated on elucidating the toxicological mechanisms of acrylamide in food. However, investigations into the environmental impact of acrylamide present in food waste are relatively scarce. Moreover, studies on the biological treatment of food waste, especially in the context of acrylamide-containing waste, are even more limited. BSFL have the ability to convert various organic wastes, such as food waste, animal manure, feces, and agro-industrial by-products, into insect biomass and a compost-like residue (a mixture of frass and substrate) [29]. Therefore, investigating the role of acrylamide in the BSFL cycle is crucial for both insect production and the food safety of insect-based products. Thus, the present study systematically investigated the multifaceted impacts of acrylamide on the growth, neurobehavioral responses, gut integrity, microbial composition, and toxicokinetics of BSFL.

The growth inhibition observed in BSFL exposed to acrylamide (Figure 1A,B) is consistent with previous research on contaminants like polycyclic aromatic hydrocarbons (PAHs) and antibiotics [15,17]. These studies also reported that such contaminants delay larval development and reduce biomass accumulation. The delayed growth peaks and decreased maximum weights of BSFL exposed to acrylamide (6.17–76.01% lower compared to the control group) suggest that acrylamide might disrupt nutrient absorption or metabolic efficiency. Notably, the strong positive correlation between larval weight and length (correlation coefficient r = 0.9359, *p* < 0.0001) indicates a systemic growth retardation rather than isolated morphological alterations. As a crucial organ for digestion and nutrient assimilation in insects, the midgut is highly susceptible to chemical stressors. Existing studies on various animal models have demonstrated that acrylamide intake impacts the composition of the gut microbiota and the integrity of the intestinal barrier [30]. For example, acrylamide can increase intestinal permeability, downregulate the expression of tight-junction protein (Occludin), and elevate the lipopolysaccharide (LPS) content in the intestine and serum. Concurrently, the expression of pro-inflammatory cytokines such as IL-6 and IL-1β is significantly upregulated [31]. Moreover, as observed in murine models, acrylamide likely disrupts tight junctions or induces apoptosis in enterocytes [9]. In this study, Trypan blue staining revealed a dose-dependent increase in midgut cell damage (2.22% in the control group vs. 25.56% at 5 mg/kg; Table S1; Figure 3), indicating that acrylamide undermines intestinal integrity. The intestinal epithelial cells and endothelial cells form a physical barrier that is crucial for intestinal homeostasis, protecting the organism from the external environment and harmful luminal products [9]. Therefore, we hypothesize that damage to intestinal cells exerts a direct impact on the growth of BSFL. Another possible mechanism is acrylamide-induced oxidative stress, which may deplete the energy reserves necessary for growth. Acrylamide is known to generate reactive oxygen species (ROS) in mammalian systems, leading to mitochondrial dysfunction [7]. If similar processes occur in BSFL, the accumulation of ROS could impair ATP production, thus slowing down biomass accumulation. However, the speculations require further verification in future experiments based on molecular biology or biochemistry.

Neurobehavioral impairments, as evidenced by a reduced crawling speed and abnormal movement patterns in BSFL exposed to acrylamide at concentrations of ≥0.5 mg/kg (Figure 2A,B), highlight the neurotoxic potential of acrylamide in insects. The decrease in peristaltic contractions (42–56 contractions/min in acrylamide-exposed groups compared to 59 contractions/min in the control group) (Figure 2C) further corroborates this conclusion. These findings are consistent with the neurotoxicity mechanisms observed in vertebrates [5]. Notably, our study is the first to demonstrate similar neurotoxic effects in BSFL, thus expanding the spectrum of organisms sensitive to acrylamide. The possible mechanism underlying acrylamide’s neurotoxic effects might be its disruption of axonal transport and synaptic function. This occurs through the formation of adducts with cytoskeletal proteins, as reported by LoPachin et al. [5]. Additionally, previous studies [32] have shown that acetylcholine (ACh) is one of the major neurotransmitters in insects. Acrylamide may inhibit the activity of acetylcholinesterase (AChE) or disrupt voltage-gated sodium channels, which, in turn, impairs neuromuscular coordination. However, to fully elucidate this mechanism, future studies should quantitatively analyze AChE activity and neuronal apoptosis in acrylamide-exposed larvae. Acrylamide-induced gut barrier dysfunction could allow microbial metabolites or inflammatory mediators to enter the hemolymph, potentially affecting the central nervous system indirectly. In our study, preliminary evidence for such cross-talk in insects was observed, as alterations in the gut microbiota (e.g., enrichment of *Enterococcus*) coincided with behavioral deficits. To validate this hypothesis, future research should explore specific neuroinflammatory pathways or neuronal damage markers. In general, Sublethal behavioral changes, like those induced by acrylamide in BSFL, may decrease the efficiency of larvae in waste processing or their ability to evade predators. Barber and LoPachin conducted a 28-day neurotoxicity study in Sprague Dawley (SD) rats exposed to acrylamide (ACR) at a dose of 50 mg/kg/day, observing significant weight loss and gait abnormalities. Consistent with these findings, Zhao et al. mentioned analogous neurotoxic outcomes in male Wistar rats subjected to lower ACR doses of 20 and 40 mg/kg/day over an extended eight-week period [7]. These emphasize the need for a more comprehensive understanding of the impact of neurotoxicants on insect physiology.

The perturbation of gut microbial communities under acrylamide stress represents a crucial adaptive response. Exposure to acrylamide significantly altered the diversity and composition of the gut microbiota in BSFL. Specifically, species richness, as indicated by the ACE and Chao1 indices, increased at a concentration of 5 mg/kg (Figure 4A). Moreover, NMDS results clearly showed sample separation between the CK and acrylamide-contaminated groups (Figure 4C). The enrichment of pathogenic genera such as *Escherichia-Shigella* and the suppression of beneficial taxa like *Klebsiella* and *Pseudomonas* [33,34] suggest that selective pressure favors acrylamide-tolerant microbes (Figure 5B). *Enterococcus*, known for its resistance to environmental stressors [35], may take advantage of acrylamide-induced dysbiosis to dominate the gut niche, potentially exacerbating intestinal inflammation. Conversely, the decline in *Klebsiella*, a genus associated with the nitrogen cycle [29], could impede larval biomass accumulation, thereby contributing to growth retardation. The reduced abundance of *Pseudomonas*, which often exhibits detoxification capabilities [36,37], might further weaken the larvae’s ability to metabolize acrylamide. These changes are similar to acrylamide-induced dysbiosis in mice, where a reduction in *Lactobacillus* and an increase in *Enterobacteriaceae* exacerbate intestinal permeability [9]. Functional profiling via KEGG pathways further validates these shifts. The analysis reveals a decrease in metabolic and immune-related pathways (Figure 6). For example, the downregulation of xenobiotic biodegradation pathways indicates a compromised detoxification capacity. Additionally, the suppression of lipid metabolism, carbohydrate metabolism, amino acid metabolism, and energy metabolism pathways aligns with the observed growth deficits (Figure S1). Notably, *Paenibacillus* was negatively correlated with axon regeneration. Collectively, these microbial and functional alterations collectively suggest that acrylamide disrupts the symbiotic relationship between BSFL and their gut microbiota, compromising host resilience.

The data presented in Figure 7 and Table 1 regarding acrylamide in BSF larvae provide valuable insights into the accumulation and elimination of this contaminant in this edible insect. The observed trends suggest that BSF larvae are capable of accumulating acrylamide from the substrate during the uptake phase, and this accumulation is influenced by the acrylamide concentration in the substrate. The differences in K_1_ values indicate that the uptake efficiency of acrylamide by BSF larvae varies with substrate concentration. Higher K_1_ values at lower acrylamide concentrations (0.05 and 0.5 mg/kg) suggest a relatively more efficient uptake process. The elimination rate constant (K_2_) also exhibits variation, with distinct values corresponding to different acrylamide concentrations, indicating that the elimination of acrylamide is concentration-dependent. The kinetic bioaccumulation factor (BAF_kinetic_) values (18.67 at 0.05 mg/kg vs. 2.90 at 5 mg/kg) and half-life for acrylamide elimination (DT_50_) values further confirm the distinct accumulation and elimination characteristics. The relatively short DT_50_ values, particularly at acrylamide concentrations of 0.05 and 0.5 mg/kg (3.25 days for 0.05 mg/kg and 1.81 days for 0.5 mg/kg), suggest that BSF larvae can eliminate acrylamide from their bodies relatively rapidly. However, at a higher acrylamide concentration of 5 mg/kg, the DT_50_ is significantly longer (8.22 days at 5 mg/kg), indicating a slower elimination process. Compared with previous studies on other contaminants in BSF or other edible insects [16,26], this research on acrylamide offers unique information regarding its toxicokinetics in BSF larvae. Nevertheless, several aspects remain to be further investigated. The underlying physiological and biochemical mechanisms responsible for the observed differences in uptake and elimination rates are not yet clear. For instance, the role of specific metabolic pathways or transporters in acrylamide uptake and elimination requires exploration. From a food safety perspective, these findings are of great significance. The accumulation of acrylamide in BSF larvae raises concerns about its potential transfer through the food chain if BSF-based products are used in food or feed applications. Understanding the toxicokinetics of acrylamide in BSF larvae is crucial for assessing the safety of these products and developing appropriate risk management strategies. The relatively short DT_50_ values at lower acrylamide concentrations, however, imply that a brief depuration period might be adequate to decrease acrylamide levels to a safe range. Future research should concentrate on identifying the optimal depuration conditions for acrylamide in BSF larvae. Given the absence of established regulations on acrylamide limits in insects as feed/food, these findings warrant further investigation, particularly aligning with EU Commission Regulation (EU) 2017/2158 for acrylamide control. The current study suggests integrating short-term elimination/depuration in clean standard substrates post-exposure to undefined waste substrates (e.g., manure or food waste) into insect-rearing GMP/HACCP protocols, rather than revising current legislation. Furthermore, investigations into the potential synergistic effects of acrylamide with other contaminants in BSF larvae, as well as the impact on the overall quality and safety of insect-based products, are necessary. During the period from 2005 to 2025, the amount of food waste in Asian countries will increase from 278 million tons to 416 million tons [11]. The application of insects for food waste treatment is bound to become increasingly widespread. These studies will contribute to a more thorough understanding of the safety of BSF as an edible insect. Such knowledge is crucial for establishing more effective regulatory guidelines for the insect-farming industry, which can ensure the safe production and utilization of insect-based food and feed products. This, in turn, will promote the sustainable development of the insect-based agriculture sector and safeguard public health.

## 5. Conclusions

This study comprehensively investigated the effects of acrylamide on BSFL, uncovering novel insights into its impact on larval growth and physiology. The results demonstrated that acrylamide exposure significantly influenced BSFL development. It postponed the growth peak and reduced the maximum weight, with the difference in weight reduction reaching 6.17–76.01% compared to the control. Moreover, acrylamide caused neurobehavioral impairments, as indicated by the deviation of crawling tracks and reduced crawling speed at higher concentrations. The gut microbiota was also disrupted, with an enrichment of pathogenic genera and a decrease in beneficial ones, along with a reduction in metabolic and immune-related functions. These findings expand our understanding of the complex physiological responses of BSFL to acrylamide.

From the perspective of food safety, this research is of great significance. Given the potential use of BSFL in waste valorization and insect-based feed production, the bioaccumulation of acrylamide in BSFL, which showed a concentration-dependent pattern, poses risks to the circular bioeconomy and food safety. The high bioaccumulation factors at lower acrylamide concentrations suggest that even low-level contamination in substrates can lead to significant acrylamide accumulation in BSFL.

## Figures and Tables

**Table 1:** Uptake and elimination kinetic parameters for acrylamide in BSFL exposed to 0.05, 0.5, and 5 mg kg^−1^ substrate (dry weight).

Acrylamide Concentration (mg/kg)	0.05	0.5	5
K_growth_ (g^−1^)	0.3891	0.3376	0.3501
K_1_ (g_substrate_$g_{\mathrm{BSFL}}^{-1}$day^−1^)	11.531	9.341	1.363
K_2_ (day^−1^)	0.163	0.182	0.084
BAF (g_substrate_$g_{\mathrm{BSFL}}^{-1}$)	18.67	15.46	2.90
DT_50_ (days)	3.25	1.81	8.22

Note: K_1_ (uptake rate constant) and K_2_ (elimination rate constant) were estimated, considering a one-compartment model assuming growth dilution. DT_50_ (half-life time for acrylamide elimination depuration period) and bioaccumulation factor (BAF) were derived from Equations (3) and (4), respectively.

## Data Availability

Summarized data are presented and available in this manuscript, and the rest of the data used and/or analyzed are available from the corresponding author upon reasonable request and pending agreement by co-authors.

## Supplementary Materials

The following supporting information can be downloaded at: https://www.mdpi.com/article/10.3390/insects16060585/s1, Figure S1: Correlations of insect gut bacteria (at the genus level with relative abundance of top 30) and predicted gut microbial functions within the organismal cluster (A), within the metabolism/cellular processes/genetic information processing/environmental information processing (B), based on Spearman’s correlation coefficients; Table S1: The staining ratio of third instar BSFL from different treatments.
