# Supplementary material for: Acrylamide Impacts on Black Soldier Fly Larvae: Growth, Toxicity, Microbes, and Bioaccumulation Risks for Food/Feed Safety
Source: Insects. 2025 Jun 1;16(6):585. doi: 10.3390/insects16060585 (PMC12192873; doi:10.3390/insects16060585)
Supplement: Supplementary file 1 [file insects-16-00585-s001.zip › insects-3617039-supplementary.pdf]

(A)

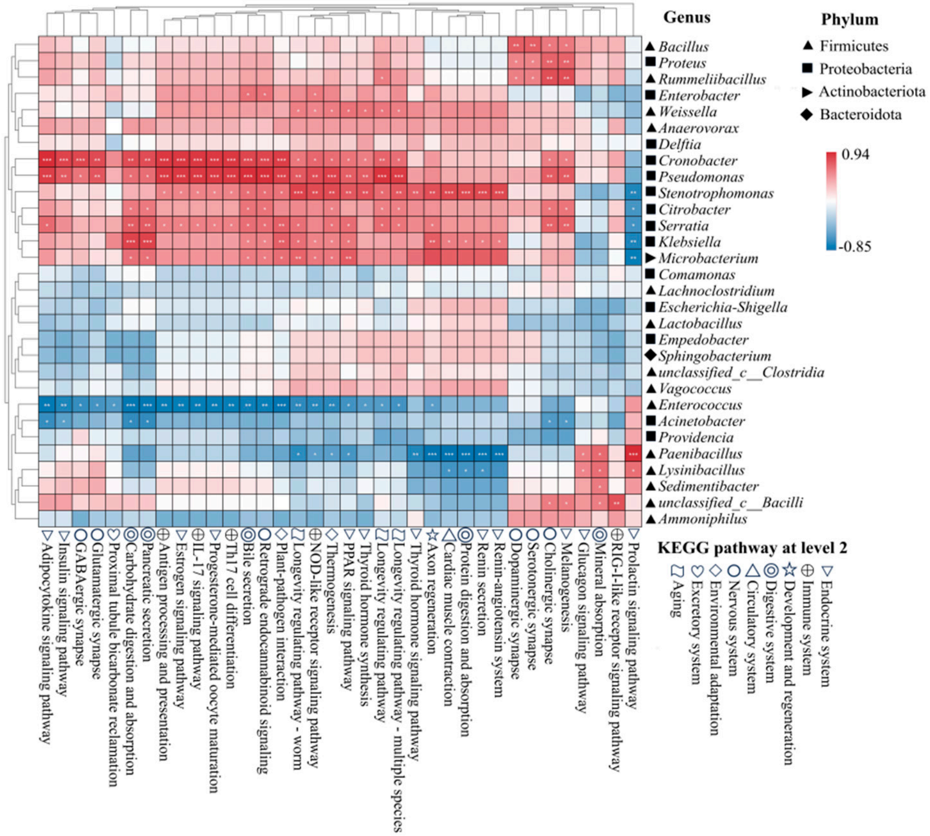
Genus
Phylum
Firmicutes
Proteobacteria
Actinobacteriota
Bacteroidota
0.94
-0.85
Bacillus
Proteus
Rummeliibacillus
Enterobacter
Weissella
Anaerovorax
Delftia
Cronobacter
Pseudomonas
Stenotrophomonas
Citrobacter
Serratia
Klebsiella
Microbacterium
Comamonas
Lachnoclostridium
Escherichia-Shigella
Lactobacillus
Empedobacter
Sphingobacterium
unclassified_c__Clostridia
Vagococcus
Enterococcus
Acinetobacter
Providencia
Paenibacillus
Lysinibacillus
Sedimentibacter
unclassified_c__Bacilli
Ammoniphilus
Adipocytokine signaling pathway
Insulin signaling pathway
GABAergic synapse
Glutamatergic synapse
Proximal tubule bicarbonate reclamation
Carbohydrate digestion and absorption
Pancreatic secretion
Antigen processing and presentation
Estrogen signaling pathway
IL-17 signaling pathway
Progesterone-mediated oocyte maturation
Th17 cell differentiation
Bile secretion
Retrograde endocannabinoid signaling
Plant-pathogen interaction
Longevity regulating pathway - worm
NOD-like receptor signaling pathway
Thermogenesis
PPAR signaling pathway
Thyroid hormone synthesis
Longevity regulating pathway
Longevity regulating pathway - multiple species
Thyroid hormone signaling pathway
Axon regeneration
Cardiac muscle contraction
Protein digestion and absorption
Renin secretion
Renin-angiotensin system
Dopaminergic synapse
Serotonergic synapse
Cholinergic synapse
Melanogenesis
Glucagon signaling pathway
Mineral absorption
RIG-I-like receptor signaling pathway
Prolactin signaling pathway
KEGG pathway at level 2
Aging
Excretory system
Environmental adaptation
Nervous system
Circulatory system
Digestive system
Development and regeneration
Immune system
Endocrine system

(B)

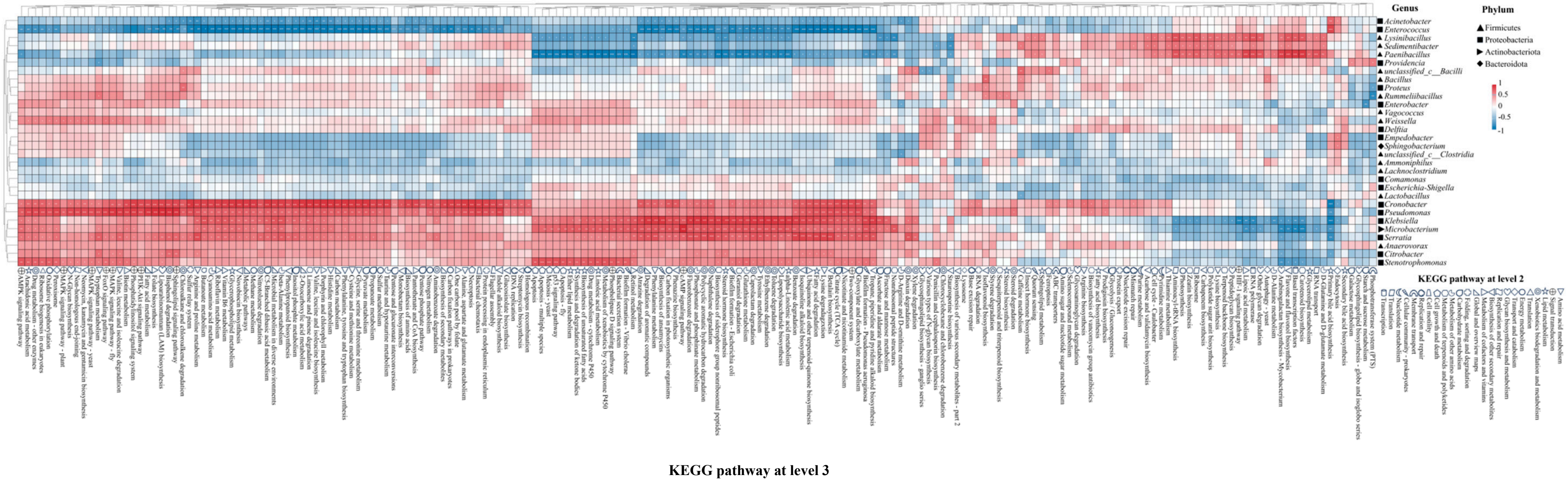

Figure S1. Correlations of insect gut bacteria (at the genus level with relative abundance of top 30) and predicted gut microbial functions within the organismal cluster (A), and within the metabolism/cellular processes/genetic information processing/environmental information processing (B), based on Spearman's correlation coefficients. "***" indicate significance at $P < 0.001$. The red squares represent a positive correlation, the blue squares represent a negative correlation, and the color depth reflects the absolute value of the correlation coefficient.

Table S1. The staining ratio of third instar BSFL from different treatments.

**Treatment**	**CK**	**0.05mg/kg**	**0.5mg/kg**	**5mg/kg**
Number of stained larvae	2	15	19	24
Total number of larvae	90	90	90	90
Staining ratio (%)	2.22	16.67	21.1	25.56
